# Second allogeneic stem cell transplantation can rescue a significant proportion of patients with JMML relapsing after first allograft

**DOI:** 10.1038/s41409-023-01942-4

**Published:** 2023-02-23

**Authors:** Luca Vinci, Christian Flotho, Peter Noellke, Dirk Lebrecht, Riccardo Masetti, Valerie de Haas, Barbara De Moerloose, Michael Dworzak, Henrik Hasle, Tayfun Güngör, Jan Starý, Dominik Turkiewicz, Marek Ussowicz, Cristina Diaz de Heredia, Jochen Buechner, Kirsi Jahnukainen, Krisztian Kallay, Ivana Bodova, Owen P. Smith, Marco Zecca, Dorine Bresters, Peter Lang, Tania Nicole Masmas, Roland Meisel, Herbert Pichler, Miriam Erlacher, Gudrun Göhring, Franco Locatelli, Brigitte Strahm, Charlotte M. Niemeyer, Ayami Yoshimi

**Affiliations:** 1 Department of Pediatrics and Adolescent Medicine, Division of Pediatric Hematology and Oncology, Medical Center, Faculty of Medicine, University of Freiburg, Freiburg, Germany; 2 German Cancer Consortium (DKTK), Heidelberg and Freiburg, Freiburg, Germany; 3 Pediatric Oncology and Hematology, IRCCS Azienda Ospedaliero-Universitaria di Bologna, Bologna, Italy; 4 Princess Maxima Center, Diagnostic Laboratory/DCOG Laboratory, Utrecht, The Netherlands; 5 Department of Pediatric Hematology-Oncology and Stem Cell Transplantation, Ghent University Hospital, Ghent, Belgium; 6 Department of Pediatrics, St. Anna Children’s Hospital, Medical University of Vienna, Vienna, Austria; 7 Department of Pediatrics, Aarhus University Hospital, Aarhus, Denmark; 8 Department Haematology, Oncology, Immunology, Gene-Therapy and Stem Cell Transplantation, University Children’s Hospital, Zurich, Switzerland; 9 Department of Pediatric Hematology and Oncology, Charles University and University Hospital Motol, Prague, Czech Republic; 10 Department of Pediatric Oncology/Hematology, Skåne University Hospital, Lund, Sweden; 11 Department of Pediatric Hematology and Oncology, BMT Unit CIC 817, Wroclaw Medical University, Wroclaw, Poland; 12 Pediatric Oncology and Hematology Service, Hematopoietic Stem Cell Transplantation Section, Vall d’Hebron University Hospital, Barcelona, Spain; 13 Department of Pediatric Hematology and Oncology, Oslo University Hospital, Oslo, Norway; 14 Division of Hematology-Oncology and SCT, New Children´s Hospital, University of Helsinki and Helsinki University Hospital, Helsinki, Finland; 15 Department of Pediatric Hematology and Stem Cell Transplantation, Central Hospital of Southern Pest—National Institute of Hematology and Infectious Diseases, Budapest, Hungary; 16 Bone Marrow Transplantation Unit, Department of Pediatric Hematology and Oncology, National Institute of Children’s Diseases, Bratislava, Slovakia; 17 Pediatric Haematology, Our Lady’s Children’s Hospital, Dublin, Ireland; 18 Paediatric Haematology and Oncology, Fondazione IRCCS Policlinico San Matteo, Pavia, Italy; 19 Stem Cell Transplantation Unit, Willem-Alexander Children’s Hospital, Leiden University Medical Center, Leiden, The Netherlands; 20 Department of Stem Cell Transplantation, Princess Máxima Centre, Utrecht, The Netherlands; 21 Department of Hematology/Oncology and General Pediatrics, Children’s University Hospital, University of Tübingen, Tübingen, Germany; 22 Pediatric hematopoietic stem cell transplantation and immunodeficiency, The Child and Adolescent Department, Rigshospitalet, Copenhagen University Hospital, Copenhagen, Denmark; 23 Division of Pediatric Stem Cell Therapy, Department for Pediatric Oncology, Hematology and Clinical Immunology, Medical Faculty, Heinrich-Heine University, Düsseldorf, Germany; 24 Institute of Cell and Molecular Pathology, Hannover Medical School, Hannover, Germany; 25 Department of Pediatric Hematology and Oncology, Bambino Gesù Children’s Hospital, Catholic University of the Sacred Heart, Rome, Italy

**Keywords:** Myeloproliferative disease, Paediatrics

## To the Editor:

Juvenile myelomonocytic leukemia (JMML) is a rare myeloproliferative disease of early childhood [1]. More than 90% of patients harbor mutations in *PTPN11*, *KRAS*, *NRAS*, *CBL*, or *NF1*. For most patients, allogeneic hematopoietic stem cell transplantation (HSCT) is the only curative therapy, while relapse is the major cause of treatment failure recorded in about 35% of patients [2, 3]. Age ≥2 years, high hemoglobin F (HbF), secondary clonal aberrations, and DNA hypermethylation are associated with an increased risk of relapse [4–6]. Treatment options for recurrent JMML are limited; for patients still on immunosuppressive therapy, discontinuation of all immunosuppressing agents is generally the first intervention. Donor leukocyte infusions (DLI) can induce a response in some relapsed patients, but the overall outcome of DLI as single strategy has been unfavorable [7]. Few data are available on the efficacy and safety of second HSCT in JMML [8]. We analyzed the outcome of 68 children with JMML who relapsed after first HSCT and received a second allograft.

A total of 434 patients with JMML registered in the European Working Group of Myelodysplastic Syndromes in Childhood (EWOG-MDS)-98/-2006 studies (ClinicalTrials.gov: NCT00047268/NCT00662090) underwent HSCT between July 1988 and January 2020. The study protocols were approved by the ethics committees of the respective institutions. Written informed consent was obtained from patients’ guardians. Of the 434 patients, 137 patients (32%) relapsed after first HSCT at a median time of 213 (range, 17–1205) days, and 78 of them (57%) received a second allograft (Supplementary Fig. 1). Most patients who did not receive a second HSCT died of disease (*n* = 49/59, overall survival at 5 years being 9%, Supplementary Figs. 1, 2). After the exclusion of 10 patients for insufficient data, 68 patients were included in this analysis. Outcome data of 25 patients included in this study was previously reported [8]. The median age of the 68 patients at second HSCT was 4.6 years (range, 1.0–15.8) (Table 1). Most patients (69%) harbored a somatic *PTPN11* mutation (Table 1). The methylation status was analyzed as previously reported in 30 patients [9], and as expected most patients (*n* = 24, 80%) showed a high methylation pattern (Table 1). Of the 68 patients, 13 were treated with DLI before the second HSCT and none of them achieved remission. Nine patients received 1 to 6 cycles of azacitidine; one patient reached a clinical complete response after 6 cycles of therapy, 3 patients obtained a partial response after 3 to 5 cycles and 5 patients experienced disease progression during treatment. For second HSCT, 16 patients were transplanted from an HLA-matched sibling, 9 from a haplo-identical donor and 43 from an unrelated donor (Supplementary Table 1). In 31 cases, the same donor was used for both allografts. For the first HSCT, most patients had received a bone marrow graft and busulfan, cyclophosphamide and melphalan (Bu/Cy/Mel) as conditioning therapy. The preparative regimen for the second HSCT was based on total-body-irradiation (TBI) in 28 patients (41%), a combination of treosulfan, fludarabine and thiotepa (Treo/Flu/TT) in 14 patients (21%), or fludarabine, thiotepa and melphalan (Flu/TT/Mel) in 9 cases (17 other regimens). Sixty-one patients (90%) achieved engraftment and one of them experienced secondary graft failure. The incidence of grade II–IV and III–IV acute graft-versus-host disease (GvHD) was 52 and 22%, respectively; chronic GvHD occurred in 37% of patients (Supplementary Fig. 3) and infection was the most common complication (Supplementary Table 2). Overall, 28 patients (41%) were alive after second HSCT with a median follow-up of 7.7 years (range, 0.4–28.3). The 5-year overall survival and disease-free-survival (DFS) were 40% (27–53%) and 36% (24–48%), respectively (Supplementary Fig. 4A). Forty patients died; relapse after the second HSCT was the main cause of death (*n* = 23), while 17 patients died of transplantation-related causes (Supplementary Table 3). The 5-year cumulative incidence of non-relapse mortality (NRM) and relapse were 23% (15–35%) and 41% (31 to 55%), respectively (Supplementary Fig. 4B). In the univariate analysis, older age at second HSCT (≥3 years) tended to be associated with an inferior DFS (HR 1.1 [0.99–1.21], *p* = 0.10) (Supplementary Table 4).The presence of a *PTPN11* mutation did not correlate with a worse prognosis. While 37% (9/24) of patients with a high methylation class experienced a relapse after the second allograft, none of the 6 patients with intermediate or low methylation pattern relapsed. Patients who suffered from an early relapse (<180 days after first HSCT) tended to have an inferior DFS (HR 0.6 [0.32–1.10, *p* = 0.10]. In the EWOG-MDS studies, the standard preparative regimen for first HSCT in JMML consists of Bu/Cy/Mel [2]. For second HSCT, many investigators had applied a TBI-regimen [8], while a non-TBI regimen has recently been preferred for second HSCT in these young patients. The probability of DFS was similar between TBI-based preparative regimens and the most recently applied Treo/Flu/TT or Flu/TT/Mel conditioning strategies in this study. (Supplementary Fig. 5). We observed 3 cases of very late NRM > 5 years after the second HSCT in association with chronic lung disease and/or chronic GvHD and/or in patients who had received a TBI-based regimen for second HSCT. Further studies are necessary to evaluate whether such late effects might be reduced by use of non-TBI regimens. The change of donor between the two allografts did not affect efficacy. There was no impact of aGvHD on DFS (grade II–IV: HR 1.3 [0.70–2.58], *p* = 0.37, grade III–IV: HR 1.7 [0.80–3.48], *p* = 0.18, *n* = 61 under risk). Similarly, there was no difference in DFS between patients who did or did not develop cGvHD over time (HR 0.7 [0.26–1.76], *n* = 47 under risk), probably due to an insufficient anti-leukemic efficacy of limited cGvHD and increased NRM with extensive cGvHD. Indeed, all events were due to NRM in patients with extensive cGvHD (5 of 8 patients, 63%) and most were due to relapse in patients with limited cGvHD (3 of 9 patients, 33%). In a multivariate Cox analysis of DFS, older was associated with worse DFS (HR 1.1 [1.00–1.22], *p* = 0.05, Supplementary Table 5).

**Table 1:** Characteristics of patients with second HSCT.

*Patient characteristics*		
Number of patients		68
Age at diagnosis	Years, median (range)	3.0 (0.2–15.1)
Gender, no. of patients (%)		
	Male	46 (68)
	Female	22 (32)
Karyotype at diagnosis, no. of patients (%)		
	Normal	46 (68)
	Monosomy 7	14 (20)
	Other aberrations	7 (10)
	Missing data	1 (2)
Mutation group, no. of patients (%)		
	*PTPN11*	42 (62)
	*NRAS*	7 (10)
	*KRAS*	1 (1)
	*NF1*	7 (10)
	All negative^a^	4 (6)
	Missing data	7 (10)
HbF	<15%^b^	23
	≥15%^c^	34
	Missing data	11
Methylation classes (*n* = 30)	High	24
	Intermediate	4
	Low	2
Time first HSCT—relapse	Days, median (range)	247 (15–788)
Time first HSCT—second HSCT	Days, median (range)	352 (35–907)
Age at second HSCT	Years, median (range)	4.6 (1.0–15.8)
Time period of second HSCT, no. of patients (%)		
	<2003	20 (29)
	2003–2009	21 (31)
	≥2010	27 (40)

*HSCT* hematopoietic stem cell transplantation, *HbF* fetal hemoglobin.

^a^Absence of mutation in *PTPN11*, *NRAS*, *KRAS*, *NF1* or *CBL*.

^b^For patients <9 months of age, HbF level was below the age-adjusted upper limit of normal.

^c^For patients 9 months or older age, HbF level was above the age-adjusted upper limit of normal.

In conclusion, a second HSCT is feasible and can cure about one-third of patients with relapsed JMML. Because vast majority of patients who had relapse died without a second HSCT, this option should be considered for all patients with relapse. The Treo/Flu/TT preparative regimen, which EWOG-MDS currently utilizes for second HSCT, appears to be as effective as a TBI-based regimen. Further research needs to focus on pre- and post-transplantation strategies to control the nature of this aggressive neoplasm and thereby reduce relapse rates. Low-dose azacitidine has been shown to be effective and can achieve complete remission in some JMML patients prior to HSCT. Therefore, azacitidine is currently recommended as a pre-HSCT therapy [10, 11]. However, whether response to azacitidine translates into better survival rate and lower risk of relapse after HSCT needs to be evaluated further. The outcome of treatment with DLI is generally poor in relapsed JMML [7]. Further studies are warranted in order to investigate whether administration of DLI in combination with azacitidine or interferon-alpha with the goal of preventing recurrence after a second HSCT can be more effective in reducing relapse rates without increasing treatment-associated toxicity [12].

## Supplementary information

Supplemental Material

## Data Availability

The datasets generated during and/or analyzed during the current study are available from the corresponding author on reasonable request.
