# Supplementary material for: Second allogeneic stem cell transplantation can rescue a significant proportion of patients with JMML relapsing after first allograft
Source: Bone Marrow Transplant. 2023 Feb 23;58(5):607–9. doi: 10.1038/s41409-023-01942-4 (PMC10162940; doi:10.1038/s41409-023-01942-4)
Supplement: Supplementary file 1 — Supplemental Material [file 41409_2023_1942_MOESM1_ESM.docx]

**Supplemental Material**

**Second allogeneic stem cell transplantation can rescue a significant proportion of patients with JMML relapsing after first allograft**

**Vinci, et al**

**Definitions and statistical analysis**

Neutrophil and platelet engraftment were defined as the first of three consecutive days with a neutrophil count > 0.5 x 10^9^/l and an unsupported platelet count > 50 x 10^9^/l, respectively. Acute and chronic graft-versus-host-disease (GVHD) were diagnosed and scored according to standard clinical criteria (1, 2). Relapse was diagnosed and response to treatment was assessed according to previously published criteria (3). A cut off level of 15% was used to define patients with increased hemoglobin (HbF) at diagnosis; in patients younger than 9 months of age, the cut-off level was set at the age-adjusted upper normal value. Overall survival (OS) and disease-free survival (DFS) were defined as the time from second HSCT to death or last follow-up and death, second relapse or last follow-up, respectively. The probabilities of OS and DFS were estimated according to the Kaplan-Meier method and DFS was compared among groups defined by categorical variables with the two-sided log-rank test. Time-to-event outcomes for relapse and non-relapse mortality (NRM) were estimated using cumulative incidence curves with relapse and NRM as competing risks. Differences in the cumulative incidence functions among groups defined by categorical variables were compared using Gray's test. Engrafted patients were considered evaluable for acute GVHD, while those surviving at least 100 days after the allograft were evaluated for GVHD occurrence. The probability for the occurrence of acute or chronic GVHD was estimated by calculating the cumulative incidence with relapse and death as competing events. The association of continuous factors with DFS was analyzed using a univariable Cox proportional hazard model. For NRM and relapse the Fine-Gray sub-distribution hazard model was used. The results are shown as hazard ratios with confidence intervals. Continuous variables were also categorized and the probabilities of DFS, NRM, and relapse in each category were demonstrated in the suppl table 4. Association between GvHD and DFS was analyzed with univariate Cox proportional hazard model treating acute and chronic GvHD as time-dependent variables. For each outcome, univariate analyses were first done (either with log-rank/Grey tests for categorical, or Cox proportional hazard model for continuous covariates), followed by multivariate Cox-analyses that included all factors with a p value < 0.1. All P values were two-sided and values < 0.05 were considered statistically significant. Software package SPSS for Windows 28.0.1.0 including R Integration Package (IBM Corp, New York, NY) and NCSS 2004 (NSCC, Kaysville, UT) were used.

1. Przepiorka D, Weisdorf D, Martin P, Klingemann HG, Beatty P, Hows J, et al. 1994 Consensus Conference on Acute GVHD Grading. Bone Marrow Transplant. 1995 Jun 1;15(6):825–8.

2. Atkinson K, Horowitz MM, Gale RP, Lee MB, Rimm AA, Bortin MM. Consensus among bone marrow transplanters for diagnosis, grading and treatment of chronic graft-versus-host disease. Committee of the International Bone Marrow Transplant Registry. Bone Marrow Transplant. 1989 May 1;4(3):247–54.

3. Niemeyer CM, Loh ML, Cseh A, Cooper T, Dvorak CC, Chan R, et al. Criteria for evaluating response and outcome in clinical trials for children with juvenile myelomonocytic leukemia. Haematologica. 2015;100(1):17–22.

**Suppl. Table 1: Pre-HSCT therapy and transplant procedures of first and second HSCT**

| ***Therapy prior to HSCT*** | | 1 HSCT |  | 2 HSCT |  |
| --- | --- | --- | --- | --- | --- |
|  | No chemotherapy | 30 |  | 49 |  |
|  | Azacitidine | 5 |  | 9 |  |
|  | AML chemotherapy | 7 |  | 0 |  |
|  | Low-dose chemotherapy | 26 |  | 10 |  |
|  | 13-cis-retinoic acid | 3 |  | 0 |  |
|  | Donor lymphocyte infusion | - |  | 13 |  |
|  | Splenectomy | 17 |  | 2 |  |
| ***HSCT procedures*** |  |  |  |  |  |
| Donor | Sibling | 21# |  | 16 |  |
|  | Haplo-identical | 4 |  | 9 |  |
|  | Other family member | 1 |  | 0 |  |
|  | Unrelated donor | 42 |  | 43 |  |
|  | Same donor for both HSCT | - |  | 31 |  |
|  | Different donor | - |  | 37 |  |
| Stem cell source | BM | 50 |  | 36 |  |
|  | PBSC | 10 |  | 26 |  |
|  | CB | 8 |  | 6 |  |
| Preparative regimen | Bu/Cy/Mel | 54 |  | 2 |  |
|  | Other Bu based regimen | 8 |  | 6 |  |
|  | Radiation (TBI/TAI) based | 4 |  | 28 |  |
|  | Treo/Flu/TT | 1 |  | 14 |  |
|  | Flu/TT/Mel | 0 |  | 9 |  |
|  | Other | 1 |  | 9 |  |
| ATG (or other Ab) | ATG | 42 |  | 37 |  |
|  | Alemtuzumab | 1 |  | 2 |  |
|  | No Ab | 25 |  | 29 |  |
| GVHD Prophylaxis | Sibling | None | 1 | None | 1 |
|  |  | CSA | 9 | CSA | 11 |
|  |  | CSA + MTX ± steroid | 10 | CSA + MTX ± steroid * | 4 |
|  |  | FK506 + MMF | 1 |  |  |
|  | Haplo-identical donor | None | 1 | None | 1 |
|  |  | CSA | 1 | CSA | 2 |
|  |  | MMF + Steroid | 1 | CSA+MMF | 1 |
|  |  | No data | 1 | MMF ± steroid ± CY | 4 |
|  |  |  |  | CY + MMF | 1 |
|  |  | Graft manipulation | 4/4 § |  | 7/9 $ |
|  | Other family member | CSA + MTX | 1** |  | 0 |
|  | Unrelated donor | CSA | 3# | CSA | 10 |
|  |  | CSA + MTX ± steroids ± MMF ± FK506 | 33 | CSA + MTX ± MMF | 25 |
|  |  | CSA + MMF | 2 | CSA + MMF | 2 |
|  |  | CSA + Steroids | 2 | CSA + Steroid | 3 |
|  |  | FK506 + MTX | 1 | FK506 + MTX | 1 |
|  |  | No data | 1 | FK506 + Steroid | 1 |
|  |  |  |  | No data | 1 |
| HSCT: hematopoietic stem cell transplantation; AML: acute myeloid leukemia; Ara-C: cytarabine; 6-MP: 6-mercaptopurine; BM: bone marrow; PBSC: peripheral blood stem cells; CB: cord blood; TBI: total body irradiation; TAI: thoracoabdominal irradiation; Bu: busulfan; Cy: cyclophosphamide; Mel: melphalan; Treo: treosulfan; Flu: fludarabine; TT: thiotepa; doses of Treo/Flu/TT regimen: treosulfan, 14 g/m2 for 3 days, fludarabine 40 mg/m2/day for 4 days and thiotepa 8 mg/kg/d for 1 day. Tapering of CSA around day +40 in the absence of grade II-IV acute GVHD and CSA discontinuation between day + 60 and +90 in JMML patients  CSA: cyclosporine A; MTX: methotrexate; MMF: mycophenolate mofetil; CY: (post-transplant) cyclophosphamide; FK506: tacrolimus; ATG: anti-thymocyte globulin. Ab: antibody # one of them was syngeneic twin, * one patient received lymphokine-activated killer (LAK) cell, **HLA-matched mother, $ CD34 positive selection (n=4), $ CD34 positive selection (n=2), T-cell depletion (n=5) # one patient received a graft with CD3 and CD19 depletion | | | | | |

| **Suppl. Table 2: Complications after second HSCT for relapsed JMML** | |
| --- | --- |
| No. of patients | (68) |
| Infection | (41) |
| Bacterial | 22 |
| Viral* | 22 |
| Fungal  Unspecified | 7  1 |
| Hepatic VOD | 10 |
| Other hepatic dysfunctions | 9 |
| Neurological complication | 3 |
| Psychosocial problems | 1 |
| Cardio-vascular complications | 3 |
| Pulmonary complications | 12 |
| Renal insufficiency | 7 |
| Endocrine disorders |  |
| Growth | 6 |
| Thyroid | 6 |
| Gonads | 1 |
| Osteoporosis | 1 |
| Cataract | 2 |
| Hepatic veno-occlusive disease (VOD) was observed in 5 patients given a total body irradiation regimen, 4 patients given treosulfan, fludarabine and thiotepa regimen and 1 patient given fludarabine, treosulfan and melpharan regimen) * One patient developed Epstein–Barr virus-associated lymphoproliferative disease | |

**Suppl. Table 3: Causes of death**

| Relapse  Acute GVHD  Chronic GVHD  Multi-organ failure  Bacterial infection  Invasive aspergillosis  Infection of unknown origin and VOD  Chronic lung disease without prior chronic GVHD  Multi-organ failure with infection after graft failure and subsequent third HSCT | 23  3 (1 with VOD)  4  2 (1 with VOD)  2  3  1  1  1 |
| --- | --- |

GVHD: graft-versus-host disease; VOD: hepatic veno-occlusive-disease; HSCT: hematopoietic stem cell transplantation.

**Suppl. Table 4: Univariate analysis of factors possibly influencing OS, DFS, relapse and NRM in second HSCT for relapsed JMML**

|  | | | |  |  |  | |  | | |  | |  |  |
| --- | --- | --- | --- | --- | --- | --- | --- | --- | --- | --- | --- | --- | --- | --- |
|  |  | 5y-DFS | | | 5y-relapse | | | | | 5y-NRM | | | | |
| Variable | n. | % | 95% CI | *P* | % | | 95% CI | | *P* | % | | 95% CI | | *P* |
| All | 68 | 36 | [24-48] |  | 41 | | [31-55] | |  | 23 | | [15-36] | |  |
| Age at diagnosis |  |  |  |  |  | |  | |  |  | |  | |  |
| < 2 years | 22 | 52 | [30-74] | n.s. | 23 | | [11-50] | | n.s. | 25 | | [11-53] | | n.s. |
| 2 - < 4 years | 32 | 33 | [16-50] |  | 48 | | [34-70] | |  | 19 | | [9-39] | |  |
| ≥ 4 years | 14 | 21 | [0-43] |  | 50 | | [30-84] | |  | 29 | | [12-65] | |  |
| Age at 2. HSCT |  |  |  |  |  | |  | |  |  | |  | |  |
| < 3 years | 17 | 63 | [39-87] | 0.07 | 18 | | [7-51] | | 0.10 | 19 | | [7-52] | | n.s. |
| 3 - < 5 years | 30 | 30 | [13-47] |  | 49 | | [34-72] | |  | 21 | | [10-43] | |  |
| ≥ 5 years | 21 | 24 | [5-53] |  | 48 | | [30-75] | |  | 29 | | [15-56] | |  |
| Gender |  |  |  |  |  | |  | |  |  | |  | |  |
| male | 46 | 36 | [22-50] | n.s. | 40 | | [28-57] | | n.s. | 24 | | [14-40] | | n.s. |
| female | 22 | 35 | [12-58] |  | 44 | | [26-72] | |  | 21 | | [9-52] | |  |
| Karyotype |  |  |  |  |  | |  | |  |  | |  | |  |
| normal | 46 | 35 | [20-50] | n.s. | 39 | | [27-57] | | n.s. | 26 | | [15-43] | | n.s. |
| abnormal |  | *38* | *[17-59]* |  | *43* | | *[26-70]* | |  | *19* | | *[8-46]* | |  |
| monosomy 7 | 14 | 29 | [5-53] |  | 50 | | [30-84] | |  | 21 | | [8-58] | |  |
| other aberration | 7 |  |  |  |  | |  | |  |  | |  | |  |
| missing | 1 |  |  |  |  | |  | |  |  | |  | |  |
| HbF at diagnosis |  |  |  |  |  | |  | |  |  | |  | |  |
| >=15%* | 34 | 33 | [16-50] | n.s. | 36 | | [23-57] | | n.s. | 30 | | [18-51] | | 0.10 |
| <15%** | 23 | 40 | [18-62] |  | 45 | | [29-72] | |  | 15 | | [5-43] | |  |
| Missing | 11 |  |  |  |  | |  | |  |  | |  | |  |
| Mutational subtype |  |  |  |  |  | |  | |  |  | |  | |  |
| *PTPN11* | 42 | 32 | [17-47] | n.s. | 47 | | [34-65] | | n.s. | 21 | | [11-38] | | n.s. |
| *NF1, NRAS, KRAS* | 15 | 45 | [21-71] |  | 28 | | [12-65] | |  | 27 | | [12-62] | |  |
| all negative | 4 |  |  |  |  | |  | |  |  | |  | |  |
| missing data^#^ | 7 |  |  |  |  | |  | |  |  | |  | |  |
| Interval 1. HSCT - relapse |  |  |  |  |  | |  | |  |  | |  | |  |
| < 180 days | 35 | 24 | [9-39] | 0.06 | 47 | | [33-67] | | n.s. | 29 | | [17-49] | | n.s. |
| ≥ 180 days | 33 | 49 | [31-67] |  | 35 | | [22-57] | |  | 15 | | [7-35] | |  |
| Interval relapse - 2. HSCT |  |  |  |  |  | |  | |  |  | |  | |  |
| < 90 days | 29 | 37 | [19-55] | n.s. | 35 | | [21-58] | | n.s. | 28 | | [16-51] | | n.s. |
| ≥ 90 days | 39 | 35 | [19-51] |  | 46 | | [32-65] | |  | 19 | | [10-38] | |  |
| Interval 1. HSCT - 2. HSCT |  |  |  |  |  | |  | |  |  | |  | |  |
| < 12 months | 39 | 30 | [15-45] | n.s. | 44 | | [31-63] | | n.s. | 26 | | [15-45] | | n.s. |
| ≥ 12 months | 29 | 45 | [26-64] |  | 37 | | [22-61] | |  | 18 | | [8-39] | |  |
| Time periods of 2. HSCT |  |  |  |  |  | |  | |  |  | |  | |  |
| < 2003 | 20 | 30 | [10-50] | n.s. | 40 | | [23-68] | | n.s. | 30 | | [15-59] | | n.s. |
| 2003 - 2009 | 21 | 43 | [21-65] |  | 38 | | [22-66] | |  | 19 | | [8-46] | |  |
| ≥ 2010 | 27 | 36 | [16-56] |  | 45 | | [29-71] | |  | 19 | | [8-41] | |  |
| Stem cell source (Haplo and CB excluded) |  |  |  |  |  | |  | |  |  | |  | |  |
| BM | 35 | 47 | [28-66] | n.s. | 45 | | [31-66] | | n.s. | 18 | | [9-37] | | n.s. |
| PB | 18 | 64 | [38-90] |  | 29 | | [14-60] | |  | 41 | | [23-72] | |  |
|  |  |  |  |  |  | |  | |  |  | |  | |  |
|  |  |  |  |  |  | |  | |  |  | |  | |  |
| Donor (CB excluded) |  |  |  |  |  | |  | |  |  | |  | |  |
| MSD | 16 | 38 | [14-62] | n.s. | 50 | | [31-82] | | n.s. | 13 | | [3-46] | | 0.07 |
| Unrelated donor |  |  |  |  |  | |  | |  |  | |  | |  |
| MUD (10/10) / (9/10) | 26 | 42 | [21-63] |  | 38 | | [22-64] | |  | 20 | | [9-44] | |  |
| UD (6/6) / (5/6) / (8/10) / incomplete  typing | 11 | 18 | [0-41] |  | 27 | | [10-72] | |  | 55 | | [32-94] | |  |
| Haplo identical donor | 9 | 33 | [2-64] |  | 56 | | [31-99] | |  | 11 | | [2-71] | |  |
| Same donor 1. and 2. HSCT (Haplo and CB excluded) |  |  |  |  |  | |  | |  |  | |  | |  |
| yes | 29 | 35 | [17-53] | n.s. | 34 | | [21-57] | | n.s. | 31 | | [18-53] | | n.s. |
| no | 24 | 34 | [12-56] |  | 47 | | [30-75] | |  | 19 | | [8-48] | |  |
|  |  |  |  |  |  | |  | |  |  | |  | |  |
| Conditioning for second HSCT |  |  |  |  |  | |  | |  |  | |  | |  |
| TBI | 28 | 39 | [21-57] | n.s. | 43 | | [28-66] | | n.s. | 18 | | [8-40] | | n.s. |
| Treo/Flu/TT | 14 | 46 | [17-75] |  | 33 | | [14-74] | |  | 21 | | [8-58] | |  |
| Other | 17 | 27 | [5-49] |  | 43 | | [24-75] | |  | 30 | | [15-63] | |  |
| Flu/TT/Mel | 9 | 33 | [2-64] |  | 44 | | [21-92] | |  | 22 | | [7-75] | |  |
|  |  |  |  |  |  | |  | |  |  | |  | |  |
| HSCT: hematopoietic stem cell transplantation; HbF: fetal haemoglobin; AML: acute myeloid leukemia; DLI: donor lymphocyte infusion; BM: bone marrow; PB: peripheral blood; CB: cord blood; Haplo: haploidentical donor; MSD: matched sibling donor; MUD: matched unrelated donor; UD: unrelated donor; TBI: total body irradiation; Treo: treosulfan; Flu: fludarabine; TT: thiotepa; Mel: melphalan; GvHD: graft-versus-host disease. * For patients < 9 months of age, HbF level was below the age-adjusted upper limit of normal. ** For patients < 9 months of age, HbF level was above the age-adjusted upper limit of normal. ^#^ Absence of mutation in *PTPN11*, *NRAS*, *KRAS*, *NF1* or *CBL*. | | | | | | | | | | | | | | |

**Suppl. Table 5: Multivariate analysis of factors possibly influencing disease free survival in second HSCT for relapsed JMML (Cox-model)**

|  | Relative risk (HR) | 95 CI | P |
| --- | --- | --- | --- |
| Age at 2^nd^ HSCT (years) | 1.1 | 1.00-1.23 | 0.05 |
| Time 1^st^ HSCT-Relapse (days) | 0.6 | 0.28-1.11 | 0.08 |

Factors with a p value <= 0.1 in the univariate analysis was included in the multivariate Cox-analysis.

**Suppl. Figure 1: Study cohort**

JMML: juvenile myelomonocytic leukemia, HSCT: hematopoietic stem cell transplantation, DLI: donor leukocyte infusion. Among the 59 patients who suffered a relapse and did not undergo a second HSCT, 49 patients (83%) died of disease at the last follow-up. Only 5 patients were still alive at least 2 years after the diagnosis of relapse without having a second HSCT after treatment including discontinuation of immunosuppressive therapy (n=1), intensive chemotherapy (n=2), azacitidine (n=1), re-infusion of donor peripheral blood stem cells (n=2) and/or DLI (n=2).

**Suppl. Figure 2: Overall survival (OS) after relapse in patients who did or did not receive second hematopoietic stem cell transplantation (HSCT)**


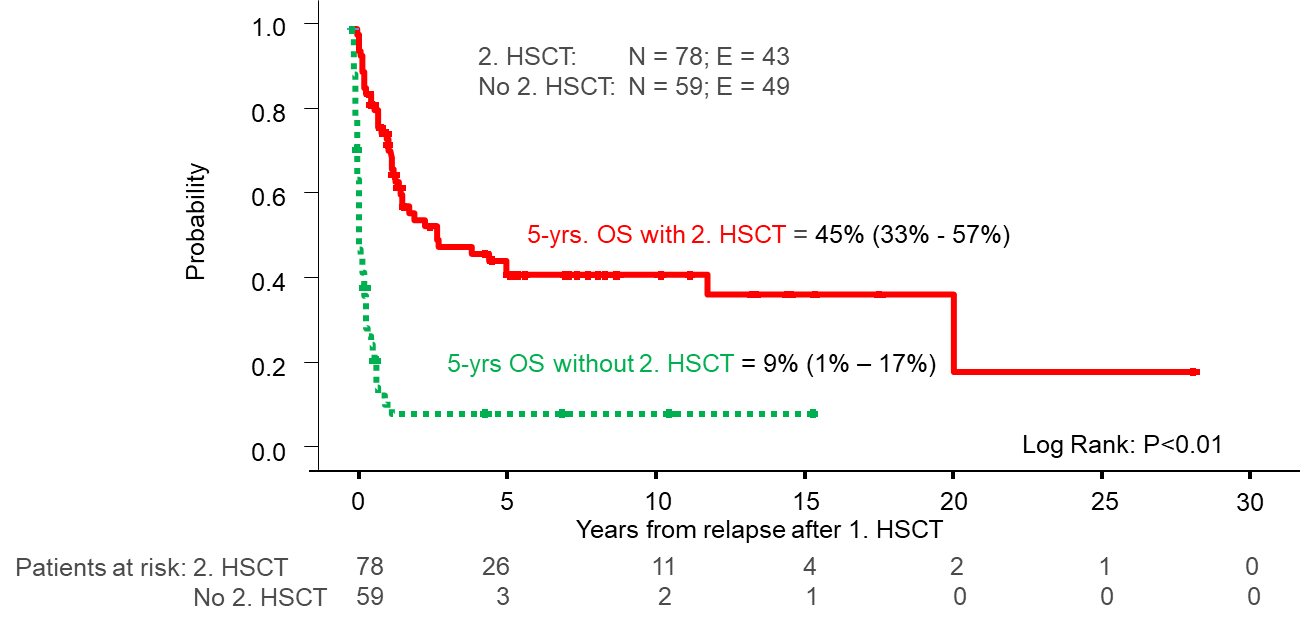


HSCT: hematopoietic stem cell transplantation; E: events; OS: overall survival

**Suppl. Figure 3: Cumulative incidence of acute and chronic graft versus host disease (GVHD) after second hematopoietic stem cell transplantation (HSCT) for relapsed JMML**

**
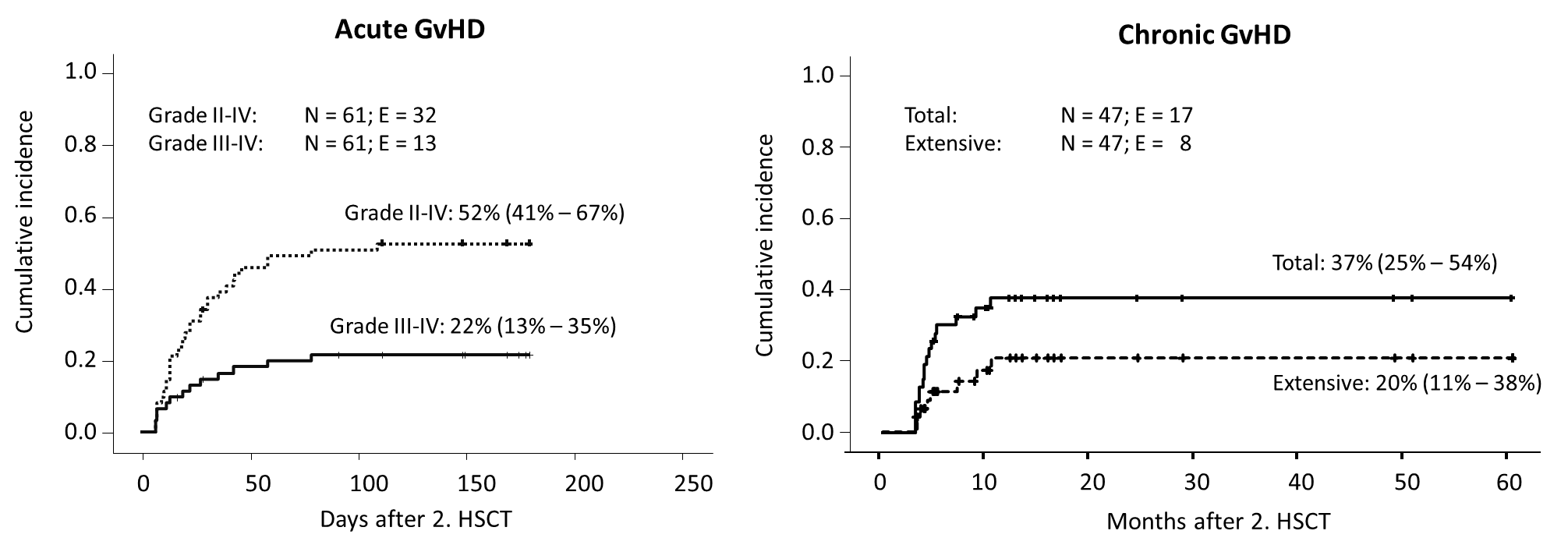
**

E: events

**Suppl. Figure 4: Outcome of patients who received a second HSCT for JMML relapse (n = 68):** Disease-free survival (A) and cumulative incidences of relapse and non-relapse mortality (B) of transplanted patients (n = 68).

**
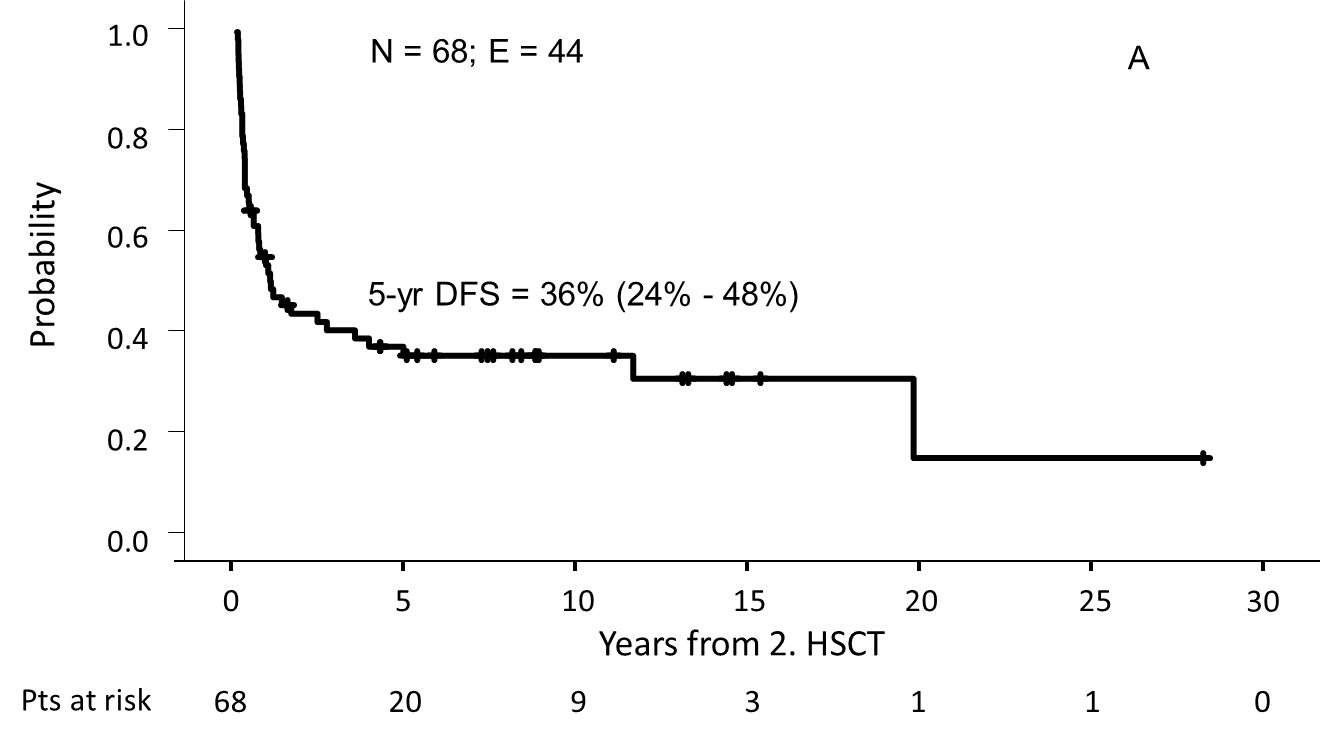
**

**
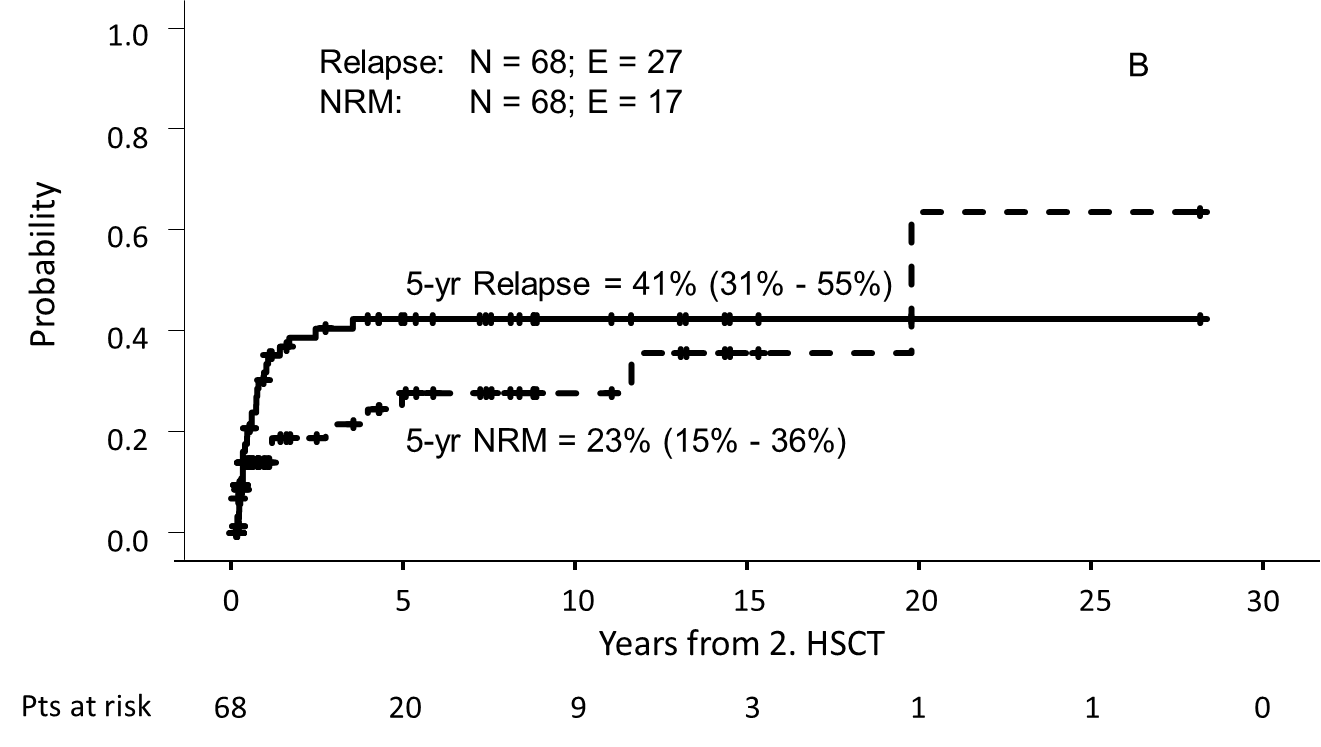
**

HSCT: hematopoietic stem cell transplantation; E: events; DFS: disease-free survival; NRM: non-relapse mortality

**Suppl. Figure 5: Disease-free survival (DFSs) after second HSCT for relapsed JMML according to conditioning regimen**

**
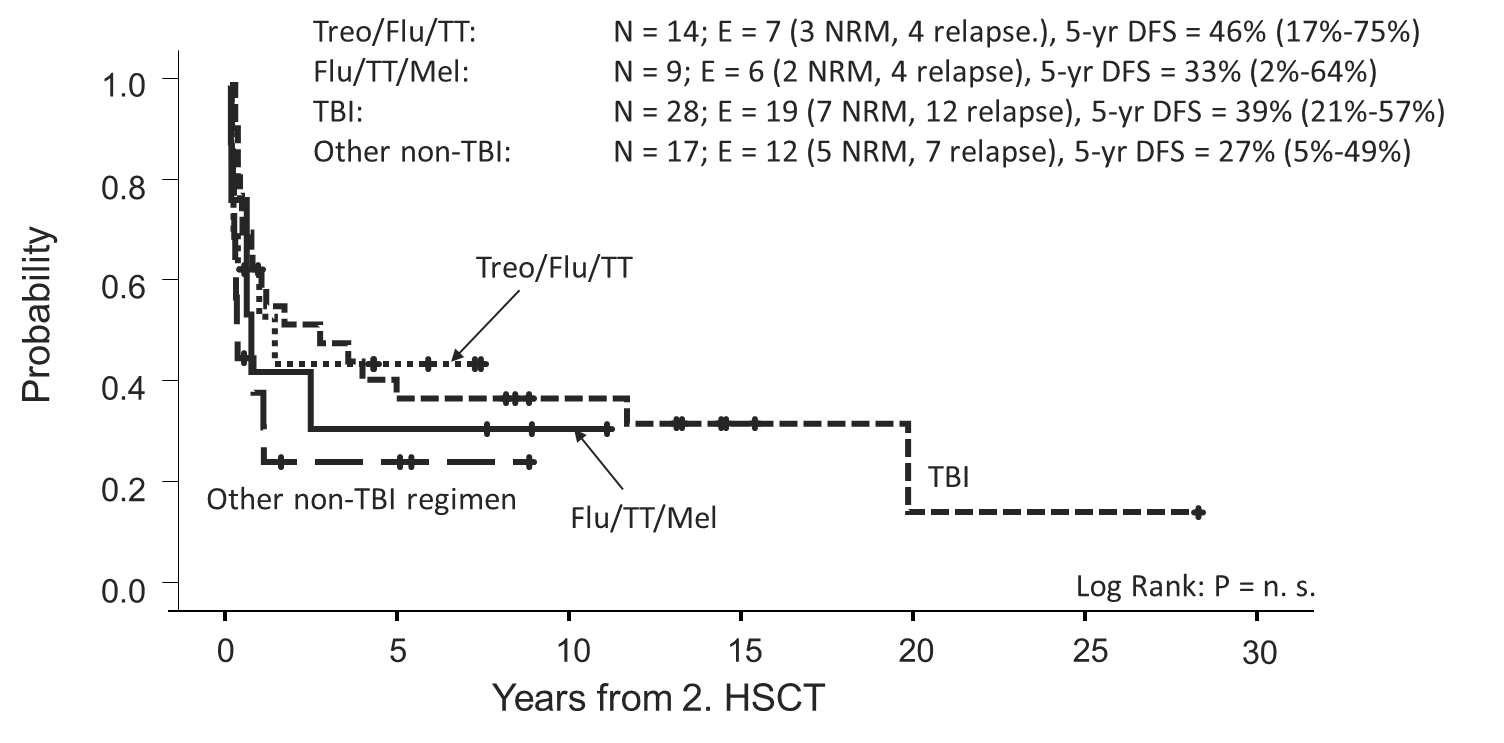
**

HSCT: hematopoietic stem cell transplantation, TBI: total body irradiation (including one with total abdominal irradiation); Treo: treosulfan; Flu: fludarabine; TT: thiotepa; Mel: melphalan; E: events, NRM: non-relapse mortality
